# Amplatzer-Based Transcatheter Paravalvular Leak Closure: A Narrative Review of Device Design, Procedural Strategy, Safety, and Outcomes

**DOI:** 10.3390/life16060905

**Published:** 2026-05-28

**Authors:** Andrei Mihnea Rosu, Claudiu Ungureanu, Emanuel Stefan Radu, Maria-Daniela Tanasescu, Eduard George Cismas, Oana Andreea Popa

**Affiliations:** 1 Department of Cardiology, Prof. Dr. Agripa Ionescu Emergency Hospital, 077015 Balotesti, Romania; andrei-mihnea.rosu@drd.umfcd.ro (A.M.R.); radu.emanuel@dcti.ro (E.S.R.); popa.oana@dcti.ro (O.A.P.); 2 Cardiovascular Department, Jolimont Hospital, Ferrer St. 159, 7100 La Louviere, Belgium; claudiu.ungureanu@helor.be; 3 Department of Semiology, Emergency University Hospital, Carol Davila University of Medicine and Pharmacy, 022328 Bucharest, Romania; 4 Department of Cardiology, “Sf. Ioan” Emergency Clinical Hospital, 061344 Bucharest, Romania

**Keywords:** paravalvular leak, transcatheter closure, Amplatzer, vascular plug, prosthetic valve, structural heart disease, hemolysis, transesophageal echocardiography

## Abstract

Paravalvular leak (PVL) is a clinically important complication of surgical prosthetic valve replacement that may lead to heart failure, hemolytic anemia, and repeat intervention. Transcatheter closure has become an accepted alternative to redo surgery in selected high-risk patients, with Amplatzer devices serving as a widely used platform because of their versatility across irregular and crescentic defects. This narrative review summarizes contemporary evidence on the role of Amplatzer-based devices in PVL closure, with an emphasis on device design, imaging-guided procedural strategies, clinical outcomes, safety, comparison with alternative occlusion systems, and future directions. Available data from registries, cohort studies, and comparative observational series indicate high technical success, meaningful improvement in symptoms and hemolysis-related burden, and acceptable medium-term durability in appropriately selected patients. Outcomes, however, remain strongly influenced by leak morphology, prosthetic valve characteristics, access route, imaging quality, and operator experience. Important limitations of the current evidence include the absence of randomized trials, nonuniform endpoint definitions, heterogeneous imaging protocols, and limited long-term comparative durability data. In contemporary structural heart practice, Amplatzer devices remain a widely used and practical platform for transcatheter PVL repair, particularly in experienced centers managing anatomically complex leaks.

## 1. Introduction

Paravalvular leak (PVL) is a well recognized complication following surgical or transcatheter valve replacement, resulting from incomplete sealing between the prosthetic device and the native annulus [1]. Reported incidence varies according to the type of prosthesis, image modality and timing of assessment. valve position ranging from 2 to 17% in surgical series to 6–32% in echocardiographic screening studies [2,3]. Clinically significant PVL most often presents with heart failure, hemolysis, or both [4]. Patients referred for percutaneous closure commonly present with advanced symptoms, often in New York Heart Association (NYHA) functional class III or IV [5], and persistent moderate-to-severe regurgitation has been associated with deterioration in ventricular function and increased mortality [6].

Surgical repair has traditionally been the standard treatment for clinically significant PVL, but repeat valve surgery is technically demanding and carries increasing risk after prior cardiac operations. Contemporary surgical series report non-negligible early mortality after redo PVL surgery, including 7% overall operative mortality in a mixed aortic/mitral cohort and 10.7% 30-day mortality in another single-center series, with mortality of 8% at the first re-sternotomy [1,2]. Observational data show that operative mortality rises with successive reinterventions and may reach 37% after a third operation [7]. Surgery remains appropriate in selected patients, particularly when there is prosthetic instability, active infective endocarditis, or anatomy unsuitable for transcatheter treatment [8]. For many patients, however, these limitations have driven the development of less invasive treatment strategies, including transcatheter PVL closure [9].

Over the past decade, percutaneous closure has become an established part of structural heart intervention in experienced centers. Contemporary series report high procedural success and acceptable safety when the anatomy is suitable, and the procedure is performed with appropriate imaging guidance and device selection [10,11]. The technical problem is straightforward to describe but often difficult to solve: most PVLs are not round defects but elongated, crescentic, oblique, or irregular channels that require a closure strategy tailored to morphology rather than nominal diameter alone [12,13].

Within this field, the Amplatzer platform merits separate review. These devices have been adopted widely because they are available in multiple configurations, can be delivered through familiar catheter-based techniques, and can be used either as single-plug solutions or in modular multi-plug strategies for more complex leaks [14]. That flexibility has made them fundamental to contemporary PVL practice, even though the evidence remains scattered across registries, single-center series, technical reports, and mixed-device cohorts.

This narrative review aims to provide a comprehensive and clinically oriented overview of Amplatzer-based transcatheter PVL closure, with emphasis on device selection, procedural strategy, clinical performance, safety, and the anatomical settings in which this platform performs best.

## 2. Literature Search Strategy

This narrative review was based on a target literature search performed in PubMed/MEDLINE, Scopus, and Web of Science, to identify relevant studies on transcatheter paravalvular leak closure with a focus on the role of Amplatzer-based devices in transcatheter PVL closure. The literature search focused primarily on studies published between January 2015 and February 2026 to reflect current structural heart practice, including advances in procedural technique, broader integration of three-dimensional transesophageal echocardiography (3D TEE) and cardiac computed tomography (CT), and contemporary use of the Amplatzer platform. Earlier landmark publications were considered selectively when needed to provide historical, technical, or conceptual background.

Articles were selected based on clinical relevance, methodological quality and contribution to procedural understanding. The search strategy combined controlled vocabulary and free-text terms related to PVL and transcatheter closure, including “paravalvular leak,” “prosthetic valve leak,” “percutaneous closure,” “transcatheter closure,” “Amplatzer,” “Amplatzer Vascular Plug,” “vascular plug II,” “vascular plug III,” and “duct occluder.” Reference lists of relevant reviews, registry reports, and original studies were also screened to identify additional eligible publications. Within this narrative review framework, titles and abstracts were first screened for relevance, and full texts were assessed when the study appeared directly applicable to the objectives of the review.

Studies were considered eligible if they involved human subjects and reported procedural, clinical, or follow-up outcomes of transcatheter PVL closure relevant to Amplatzer-based practice. Accordingly, the review included both Amplatzer-specific series and mixed-device cohorts in which Amplatzer devices represented a major component of practice or provided meaningful comparative context. Eligible study designs included multicenter registries, prospective or retrospective cohort studies, and comparative analyses. Small case reports were generally excluded unless they offered distinct technical, anatomical, or mechanistic insights directly relevant to device selection or procedural strategy. When overlapping cohorts were identified, priority was given to the most comprehensive or most recent dataset. Priority was also given to studies that were most informative for contemporary practice, particularly larger series, multicenter datasets, studies with detailed imaging and procedural reporting, and analyses with clear definitions of technical success, clinical outcomes, residual regurgitation, complications, and follow-up.

Given the narrative design of this review, no formal meta-analysis or structured risk-of-bias assessment was performed. Instead, studies were appraised qualitatively with attention to sample size, study design, imaging guidance, definitions of technical and clinical success, residual regurgitation, hemolysis-related outcomes, procedural complications, and duration of follow-up. Particular emphasis was placed on studies reflecting contemporary imaging integration and current structural heart program experience. Evidence was not weighted quantitatively, but greater interpretive emphasis was placed on studies with broader clinical applicability, contemporary procedural relevance, and more complete outcome reporting.

The final body of literature was synthesized descriptively and organized thematically around device design and selection, procedural strategy, clinical performance, safety, evidence gaps, and durability. This approach was intended to provide a clinically focused overview of the role of Amplatzer devices in the contemporary transcatheter management of PVL. The thematic synthesis was designed to integrate technical, anatomical, and outcome-oriented evidence across sections while preferentially relying on the most informative and representative studies available for each topic.

The review is not a systematic analysis and does not aim to provide a comprehensive quantitative synthesis of the literature.

## 3. Pathophysiology and Anatomical Complexity of PVL

PVL is defined as regurgitant flow between the prosthetic sewing ring and the adjacent native tissue caused by incomplete sealing of the prosthesis within the annulus [8]. Small paravalvular jets are common after valve replacement, but only a minority become clinically significant. When moderate or severe, PVL may lead to heart failure symptoms, hemolytic anemia, and progressive ventricular dysfunction.

PVL develops when the prosthetic sewing ring fails to achieve durable apposition to the native annulus. The usual substrate is partial suture dehiscence or incomplete annular sealing at the time of implantation. Several factors contribute to this process. Heavy annular calcification may prevent uniform seating of the prosthesis, friable or inflamed tissue may not provide stable support, and infective endocarditis may further weaken the annulus and promote prosthetic detachment [15]. Technical factors at surgery and distortion of annular geometry after prior cardiac operations may also create paravalvular channels through which regurgitant flow develops.

The frequency and anatomical pattern of PVL vary by valve position. Reported incidence is higher after mitral valve replacement, where PVL has been described in 7–17% of surgically implanted prostheses, compared with approximately 2–10% after aortic valve replacement. Mitral PVL is more common than aortic PVL and is usually more complex anatomically [8]. This difference reflects, at least in part, the geometry of the mitral annulus, which has a larger circumference, a saddle-shaped non-planar configuration, and dynamic motion throughout the cardiac cycle. The aortic annulus is generally more circular and structurally rigid, which favors more uniform prosthetic seating. As a result, aortic PVLs are often smaller and more localized, whereas mitral PVLs more often extend along a broader segment of the annulus and present greater technical difficulty during transcatheter closure.

From an interventional standpoint, PVL is better understood as a paravalvular tract than as a simple circular orifice. Unlike congenital septal defects, these leaks commonly follow the contour of the prosthetic ring and form crescentic, slit-like, elongated, or serpiginous channels rather than discrete round openings [16]. This morphology has direct procedural consequences. A single occluder may be sufficient for a focal defect, but longer or more irregular channels often require individualized device selection or implantation of more than one device to achieve a satisfactory result [17]. In mitral PVL, certain annular segments, particularly the anterolateral and posteromedial regions, appear more prone to leak formation, likely because of less uniform tissue support and greater mechanical stress [18].

Hemolytic anemia is one of the characteristic manifestations of clinically significant PVL. The mechanism is related to high-velocity regurgitant flow through a narrow paravalvular channel, which generates turbulent shear stress and leads to erythrocyte fragmentation. The severity of hemolysis does not necessarily correlate with leak size. Small defects may produce severe hemolysis when jet velocity is high, whereas larger defects may generate less red cell destruction if flow is distributed through a wider channel [18].

These anatomical features determine procedural strategy. Device selection and procedural planning depend on an accurate definition of the defect shape, length, orientation, and relation to prosthetic components [16,17]. Smaller and isolated defects are generally more amenable to straightforward closure, whereas larger, elongated, or irregular channels often require more complex strategies, including staged or multi-device implantation [17].

**Figure 1:**
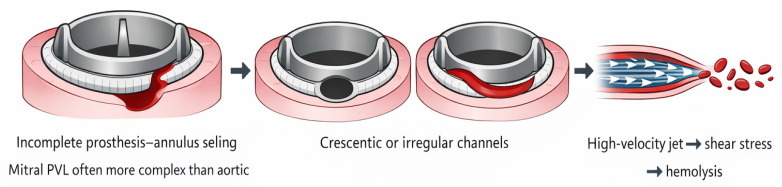
Pathophysiology and anatomical complexity of paravalvular leak. Paravalvular leaks arise from incomplete sealing between the prosthetic valve sewing ring and the native annulus, producing regurgitant channels around the prosthesis. Anatomical differences between the mitral and aortic valves influence the complexity of PVL. Defects frequently exhibit crescentic or irregular morphologies rather than circular openings, which may complicate device sealing. Narrow paravalvular channels can generate high-velocity jets associated with shear stress and hemolysis.

The principal mechanisms underlying PVL formation, the influence of valve position, defect morphology, and the hemodynamic basis of hemolysis are summarized schematically in Figure 1.

## 4. The Amplatzer Platform: Engineering Principles and Device Selection

### 4.1. Platform Overview

The Amplatzer occlusion platform is frequently used in transcatheter structural heart interventions and represents one of the principal device systems used for PVL closure. These devices are constructed from braided nitinol mesh, a nickel–titanium alloy with shape-memory and superelastic properties. This material allows the device to be compressed within a delivery catheter and to self-expand after deployment. Once released, the nitinol framework returns to its preconfigured geometry, providing mechanical stability while adapting to irregular cardiovascular defects. The mesh structure allows the occluder to conform to irregular paravalvular channels while maintaining sufficient radial force for anchoring within the defect tract [19].

A defining feature of the Amplatzer platform is controlled catheter-based deployment. During implantation, the device remains attached to a delivery cable until its position is confirmed by fluoroscopic and echocardiographic guidance. If the device interferes with prosthetic valve function or does not adequately engage the defect, it can be recaptured and repositioned before final release. This capability is particularly important in PVL closure, where defects often lie adjacent to prosthetic valve leaflets and accurate placement is required to avoid obstruction of valve motion [20].

In contrast to circular septal defects, PVLs usually form non-circular tracts along the prosthetic ring, which makes tract-filling devices more suitable than disc-based occluders in many cases [21]. Accordingly, vascular plug–type devices are commonly favored because they occupy and seal the channel itself rather than simply cover an orifice. Expansion of the braided nitinol mesh partially obstructs the leak tract, reduces high-velocity flow, and provides a scaffold for thrombus formation within the device interstices, which progressively contributes to sealing after implantation. Most Amplatzer devices used for PVL closure were originally developed for other structural or vascular indications, and their use in this setting remains largely off-label [12,21].

The Amplatzer platform includes several device configurations with different geometries and delivery characteristics. In clinical practice, a limited subset of these occluders—most commonly the Amplatzer Vascular Plug II, Amplatzer Vascular Plug III, and the Amplatzer Duct Occluder—are used for PVL closure. Device selection is guided primarily by leak morphology, the spatial relationship to the prosthetic valve, and the constraints of the chosen delivery approach.

### 4.2. Devices Used in PVL Closure

Device selection for PVL closure depends primarily on defect morphology, prosthetic valve characteristics, and the risk of interference with leaflet motion. Most PVLs are not circular. They are more often crescentic, oval, oblong, or serpiginous, which explains why different occluders perform differently across anatomies and why no single device is suitable for all defects [22,23,24]. In current practice, dedicated PVL devices and off-label Amplatzer systems are both used, with the final choice shaped by channel geometry, device deliverability, and the need for stable anchoring without compromising prosthetic valve function [3,21]. Among currently used devices, the Amplatzer Vascular Plug III (AVP III) is particularly suited to crescentic, oval, and elongated PVLs because its oblong multilobed configuration allows better adaptation to non-circular defects than cylindrical occluders. The Amplatzer Vascular Plug II (AVP II) remains one of the most adaptable off-label options. Its braided nitinol mesh, low profile, and catheter deliverability make it suitable for many focal PVL channels, particularly when the defect allows secure seating of the plug body. Small, round or short tubular leaks may be closed with a single device, whereas longer crescentic or oblong channels often require sequential or simultaneous implantation of more than one plug to improve sealing along the length of the defect [21,22]. More recent multicenter experience indicates that AVP II is still used in transcatheter PVL closure, although less frequently than AVP III or dedicated PVL devices, reflecting the growing preference for morphology-specific systems in more complex anatomies [3].

The Amplatzer Duct Occluder (ADO) and ADO II have a narrower role. Earlier experience with off-label duct occluders suggested that they may be effective for smaller, more focal leaks, particularly when the anatomy is not broad and crescentic, but mismatched device geometry may leave residual regurgitation or create interaction with adjacent prosthetic structures [23]. More recent reports support their selective use as adjunctive devices for residual focal channels after primary plug implantation, including in mitral PVL closure under detailed 2D and 3D transesophageal echocardiographic guidance [25]. Other non-dedicated Amplatzer occluders, including muscular ventricular septal defect devices, remain reasonable alternatives in selected high-risk patients, but their use is best reserved for anatomies in which anchoring and disc position can be predicted with confidence [24,26]. Overall, AVP II is better suited to focal or tubular channels, whereas duct occluders appear most useful as selective off-label or adjunctive solutions rather than routine first-line devices for complex crescentic PVLs.

Table 1 summarizes the practical morphology-based roles of the main Amplatzer devices used in PVL closure.

**Figure 2:**
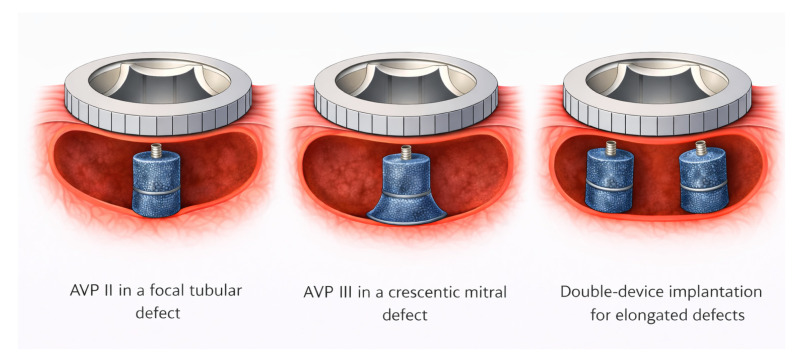
Representative morphology-based device strategies for PVL closure. (**Left**): AVP II positioned in a focal tubular defect. (**Middle**): AVP III used in a crescentic mitral PVL, reflecting its greater conformity to non-circular channels. (**Right**): double-device implantation strategy for elongated defects requiring sequential or combined occlusion along the leak tract.

The relationship between occluder geometry and PVL morphology is illustrated schematically in Figure 2.

## 5. Procedural Strategy and Imaging Guidance

Procedural success in PVL closure depends on careful anatomical definition before the procedure, thoughtful route selection, and continuous imaging guidance during crossing, device deployment, and final assessment. Contemporary practice has made PVL closure an imaging-driven intervention in which defect morphology, orientation, and the relation of the channel to surrounding prosthetic structures largely determine technical feasibility. Echocardiography, particularly transesophageal echocardiography (TEE) with three-dimensional (3D) acquisition, remains the main imaging modality. Computed tomography (CT), fusion imaging, and 3D printing may provide additional value in selected anatomically complex cases [3,27,28].

Preprocedural planning begins with characterization of the leak, including valve position, circumferential location, size, tract length, and its relation to prosthetic leaflets or discs. The FFPP registry showed that TEE was used in 96.5% of procedures and 3D echocardiography in 87.4%, reflecting the central role of 3D imaging in current PVL practice [27]. Three-dimensional TEE is particularly useful because it provides an en-face view of the prosthesis and a shared spatial reference for the operator and imaging specialist. CT can complement echocardiography by providing delineation of channel course, predicting fluoroscopic working angles, and, in selected cases, allowing fusion of preprocedural CT with live fluoroscopy to facilitate leak crossing. Cardiac magnetic resonance has a more limited role and may be considered selectively when quantification of regurgitation severity remains uncertain [27].

Advanced planning tools may be helpful in difficult anatomy. In a 166-patient series [28], acute procedural success was 92.8%, and cases guided by TEE combined with 3D printing had shorter procedure and fluoroscopy times than cases guided by transthoracic echocardiography alone. A smaller feasibility study [29] showed concordance between the preprocedurally selected and ultimately implanted occluder in 7 of 8 mitral PVL cases. Although not required routinely, 3D printing may assist selected cases with complex mitral anatomy or uncertain device-channel interaction [28,29].

The access strategy is determined mainly by the valve position and leak orientation. For mitral PVL, the transseptal antegrade route is generally the preferred approach because it provides a familiar and versatile path from the femoral vein to the left atrium, particularly for anterior or anterolateral defects. Transseptal puncture should be adapted to the leak location to create the most direct trajectory toward the defect, and 3D-TEE is helpful both for puncture guidance and for confirming that the guidewire has crossed the PVL rather than the prosthetic valve itself. Retrograde arterial access remains useful in selected mitral leaks, especially when the defect is medial or posterior, when septal crossing is unfavorable, or when additional support is needed. The transapical route is more invasive and therefore reserved for selected cases, but it remains an important bailout or, in some anatomically difficult mitral PVLs, a primary option when antegrade and retrograde trajectories are unfavorable or when large devices require a short and stable delivery course [11,27].

For aortic PVL, procedural planning is usually more straightforward. Both expert-based reports and contemporary clinical series describe retrograde arterial access as the usual approach, through either femoral or, in selected cases, radial entry depending on defect location and operator preference [11,27]. In current practice, aortic PVL closure is often technically easier than mitral closure, although large defects, angulated channels, and unfavorable prosthetic geometry may still complicate device delivery. In the single-centre series [11], technical and procedural success rates were 93.33% and 91.11%, respectively, supporting the feasibility of individualized access selection in experienced hands. These results are directionally consistent with larger registry data, although success remains anatomy-dependent and influenced by operator experience [30].

An arteriovenous wire loop is not necessary in every case, but it remains a useful adjunct when support is insufficient for sheath advancement across a resistant or angulated mitral channel. Its role is to improve support in selected cases rather than to serve as a standard component of every procedure [27]. This can be particularly helpful in long or oblique defects, or when antegrade or retrograde crossing alone does not provide an adequate delivery rail [27,28].

Several procedural pitfalls explain the need for continuous imaging guidance. The first is failure to cross the intended leak channel or inadvertent passage through the prosthetic valve. The second is malposition of the device, with a protrusion that restricts leaflet or disc motion. The third is incomplete sealing with residual regurgitation, which may require repositioning or implantation of an additional device. In the FFPP registry, echocardiography guided the leak crossing and device positioning, and allowed real-time assessment of residual PVL, prosthetic function, and the relation between the device and surrounding structures before release [3]. Before final release, imaging should confirm stable device position, preserved prosthetic leaflet or disc mobility, satisfactory reduction in regurgitation, and absence of device-related obstruction or interference with adjacent structures. Complete abolition of regurgitation is not always necessary if the residual jet is trivial and prosthetic function remains intact; by contrast, persistent hemodynamically relevant residual leak should prompt repositioning or additional closure. Important complications include device embolization, prosthetic valve dysfunction, vascular injury, stroke, persistent hemolysis, and the occasional need for urgent surgical conversion.

These procedures are best performed in experienced structural heart centres with close collaboration among interventional cardiologists, imaging specialists, anaesthesiologists, and cardiac surgeons, particularly when continuous TEE guidance is required. Registry data also show that outcomes are influenced by both anatomy and experience. In the HOLE registry, technical success was 86.6% and procedural success 73.2%, while use of the AVP III and higher centre volume predicted better success in mitral lesions [30]. Procedural strategy in PVL closure is therefore best approached as a morphology-driven and imaging-guided process in which outcomes depend on the integration of anatomical planning, route selection, catheter support, and careful intraprocedural reassessment.

## 6. Clinical Performance of Amplatzer Devices

The clinical performance of transcatheter PVL closure is best judged by four domains: procedural success, clinical benefit, hemolysis response, and durability. However, interpretation of available data requires caution, as most evidence derives from observational studies with heterogeneous definitions and patient populations.

The literature is heterogeneous, with differences in study design, valve position, device selection, and endpoint definitions, so direct comparison between cohorts remains imperfect. Even so, several patterns are consistent. Technical success is usually high, symptom burden often falls substantially after a successful procedure, hemolysis improves in many patients when the residual jet is adequately reduced, and benefit can persist for years in selected cases. Table 2 summarizes the main outcome signals from representative studies of predominantly Amplatzer-based PVL closure, with emphasis on procedural success, clinical benefit, hemolysis response, and follow-up durability.

### 6.1. Procedural Success

Procedural success with predominantly Amplatzer-based strategies is consistently high, usually in the high 80% to low 90% range across contemporary series [30,31,32,33,34,35,36,37]. The exact percentage varies less because of a major difference in technical performance than because studies define success differently. Some use device implantation as the endpoint, whereas others require a specified reduction in regurgitation or freedom from major complications. That distinction explains why the Spanish HOLE registry reported technical success of 86.6% but a lower procedural success of 73.2%, while the UK and Ireland registry reported device implantation success of 91%, and the international registry reported overall technical success of 87.4% [30,31,32]. From a clinical standpoint, these studies describe a similar reality: most procedures end with successful device delivery and a meaningful reduction in regurgitation, but the proportion meeting stricter composite definitions is lower.

A second pattern is that aortic PVL is often more straightforward than mitral PVL, although the difference is not absolute. In the international registry, technical success was 91.4% in aortic PVL and 85.0% in mitral PVL [32]. This difference likely reflects greater technical complexity in mitral interventions, although results remain strongly dependent on anatomy and operator experience.

Smaller series and more focused registries report success rates at the upper end of this range, particularly when device choice is closely matched to anatomy. The Greek multicenter cohort reported technical success of 90.0%, the tertiary-center prospective study reported technical success of 93.3% and procedural success of 91.1%, and the registry of exclusive multi-plug AVP III implantation reported technical success of 92.8% with procedural success of 89% [11,34,37]. The small series using a rectangular AVP III configuration for crescentic leaks reported procedural success in all cases, which is consistent with the principle that appropriate device geometry matters most when the defect is elongated or irregular [35]. The same applies to multi-plug strategies in anatomies that are not likely to be sealed adequately with a single round device [36,37].

The quality of the acute result matters as much as successful delivery. A device that sits well but leaves a clinically important residual jet is not a satisfactory endpoint. This is particularly relevant when hemolysis is the presenting problem. In the hemolysis-focused registry, recovery was best when the reduction in PVL cross-sectional area exceeded 90% [4]. In practical terms, procedural success should be judged by the quality of residual leak reduction rather than by implantation alone [4,34,38].

It should be emphasized that most of these data originate from experienced centers, and outcomes may not be directly generalizable to lower-volume settings.

### 6.2. Clinical Outcomes

Clinical benefit after successful PVL closure is frequently reported, particularly in terms of functional status and hemolysis burden. Improvement in heart failure symptoms is one of the most consistent findings in the literature [11,31,32,33,34,35,36]. Even when studies do not provide a uniform composite endpoint, most report a clear shift toward lower NYHA class after closure. In the UK and Ireland registry, the mean NYHA class improved from 2.7 ± 0.8 to 1.6 ± 0.8 [31]. In the international registry, clinical success was reported in 70.3% of mitral PVL cases and 88.0% of aortic PVL cases at 1 month [32]. The Greek multicenter cohort reported overall clinical success of 78.3%, with better results in patients treated primarily for heart failure than in those treated for hemolysis [34]. Smaller contemporary studies point in the same direction, with symptom improvement reported after both single-device and multi-device closure strategies [11,35,36].

Hemolysis-related benefit is more unevenly reported, but the pattern is still clear when laboratory data are provided. The strongest studies document rising hemoglobin, falling LDH, improvement in bilirubin, and reduction in transfusion requirements after successful closure [4,24,33,34,36,39]. In the hemolysis-focused registry, laboratory recovery over 6 months was most pronounced when PVL cross-sectional area was reduced by more than 90% [4]. In the all-comers registry with long follow-up, closure was associated with increased hemoglobin, lower LDH, and lower transfusion requirement [33]. The Greek cohort documented significant improvement in hemoglobin, LDH, and indirect bilirubin at 1 month [34]. In the mitral case series, LDH fell significantly, although persistent hemolytic anemia remained present in a small minority at 30 days [36]. This pattern is clinically important: hemolysis improves in many patients, but it is less forgiving of residual leak than heart failure symptoms.

This highlights an important clinical distinction: unlike heart failure symptoms, hemolysis appears to be highly sensitive to even small residual high-velocity jets.

The dedicated-device literature provides additional context for symptom and hemolysis relief, although these data should not be interpreted as direct comparisons with Amplatzer platforms. In the PLD registries, the proportion of patients in NYHA class III/IV fell markedly during follow-up, and transfusion dependence related to hemolysis also decreased substantially [24,39]. These reports are useful because they confirm the broader principle that effective PVL reduction translates into clinical benefit, regardless of the specific occluder chosen when anatomical selection is appropriate.

Rehospitalization is reported less consistently as an isolated endpoint, which limits direct comparison across studies. Even so, the overall clinical trajectory after successful closure is favorable. Improvements in functional class, lower transfusion dependence, and registry definitions of clinical success that incorporate freedom from major adverse events or heart failure readmission indicate that transcatheter PVL closure offers benefit beyond simple echocardiographic reduction in regurgitation [32,39].

### 6.3. Long-Term Durability

Durability is reported less consistently than acute procedural success, but the available data support sustained benefit in many patients, particularly when the initial result is good [5,33,34,35,37,40]. The most informative reports are those with follow-up beyond 2 years, where functional improvement, persistent reduction in leak severity, and relatively low rates of repeat intervention can be assessed with more confidence.

The strongest long-term data come from single-center series with extended follow-up. In one large mitral PVL cohort, the median follow-up was 41.8 months and the mean follow-up was 47.7 ± 35.7 months. Functional improvement persisted in 77.4% of patients at last follow-up, and repeat intervention was required in 14.6%, including 11.5% repeat percutaneous closure and 3.1% surgical reintervention [40]. Survival after the first PVL closure procedure was 75% at 1 year, 64.3% at 2 years, 51.3% at 4 years, and 45.2% at 5 years [40]. These figures need to be read in the context of an elderly, comorbid, high-risk population rather than as a measure of device failure alone.

Longer-term results from predominantly Amplatzer-based practice are supported by the all-comers registry with a median follow-up of 66 months, in which symptomatic benefit and hematologic improvement were maintained in many patients [33]. The crescentic-leak series and the AVP III multi-plug registry also reported stable medium-term results over approximately 2 years, with low rates of late clinical efficacy failure in most successfully treated patients [35,37]. In post-TAVI PVL, the PLUGinTAVI registry showed durable benefit over a mean follow-up of 21.7 ± 16.2 months, with most patients remaining in NYHA class I–II, fewer heart failure hospitalizations, and no reported reinterventions among successfully treated cases [5].

The main message from these studies should be interpreted cautiously, as durability outcomes remain influenced by patient comorbidity, anatomical complexity, and variability in follow-up reporting. Durable benefit is achievable, but it depends heavily on the quality of the initial result and the complexity of the underlying anatomy. Repeat intervention is not rare in difficult mitral cases or in high-risk populations, so durability is better understood as persistence of clinical benefit over time rather than complete freedom from later events [5,33,34,35,37,40].

### 6.4. Predictors of Success and Failure

Outcome after transcatheter PVL closure is shaped primarily by anatomy, valve position, residual regurgitation, and operator experience [23,32,40,41]. Among these, anatomical complexity remains the dominant determinant of technical and clinical success, particularly in broader or more irregular leaks that may require individualized or multi-device strategies [23].

Valve position further modifies procedural difficulty. In the Spanish HOLE registry, predictors of success differed between valve locations: higher-volume centers were associated with better mitral results, whereas leak size carried greater weight in aortic PVL. In longer-term follow-up series, mitral location has also been associated with less favorable outcomes in more complex populations [40].

Operator and center experience have a clear effect on results [32,41]. Contemporary multicenter studies show better outcomes in programs with strong imaging support, experienced structural operators, and consistent heart-team case selection. One multicenter cohort identified the learning curve as an important determinant of technical success, and the only periprocedural death occurred during the early phase of the program [41]. These data support the concentration of complex PVL closure in centers with sufficient procedural volume and imaging expertise.

Residual regurgitation after closure remains one of the strongest markers of later outcome. In earlier series, effective reduction in PVL was the only predictor associated with freedom from death, heart failure rehospitalization, or surgical revision [23]. More recent data points in the same direction. Lack of procedural success at the first intervention, multiple PVLs, hemolytic anemia as the presenting indication, and persistent functional limitation during follow-up are all associated with worse longer-term outcomes [40,41]. In practice, the best results come when the closure strategy matches the anatomy and the residual jet is reduced to the lowest level that is technically and mechanically safe.

## 7. Safety Profile and Complications

Transcatheter PVL closure with Amplatzer-based devices is a well-established alternative to repeat surgery in patients with prohibitive or high operative risk, and comparative series support its use in appropriately selected cases [42,43]. The safety profile is favorable, but it varies with anatomy, prosthetic valve type, access route, device choice, and the quality of intraprocedural imaging [43,44]. In practice, complications fall into four broad groups: device-related events, residual leak with hemolysis, access- and procedure-related complications, and less frequent but more serious events such as stroke, endocarditis, and death.

Device-related complications are among the most characteristic hazards of the procedure. These include device malposition, incomplete seating, instability during deployment, embolization, residual shunting, and interference with prosthetic leaflet or disc motion. In most cases, the underlying issue is a mismatch between the occluder and the leak channel, especially when the defect is crescentic, elongated, serpiginous, or angulated, or when its relation to the prosthetic hinge mechanism is underestimated. In one multicenter series, some devices could not be stabilized and had to be abandoned, and follow-up identified cases of occluder-related mechanical disc interference that required reoperation [45]. For that reason, apparent success at release should not be equated with durable compatibility with the prosthesis. A simulation-based analysis of complex cases showed that multimodality imaging could identify the risk of leaflet blockade, residual regurgitation, and embolization before release [46]. In day-to-day practice, device choice is driven far more by channel morphology than by nominal defect diameter.

A residual leak is both an efficacy issue and a safety issue. Mild residual regurgitation may be acceptable in selected patients, but persistent high-velocity jets can maintain or aggravate hemolysis even when the overall leak burden has been reduced. This matters particularly in patients referred for hemolytic anemia rather than heart failure. In one single-center registry, residual leakage was absent or mild in 80% of cases, yet 20% of patients still had moderate-to-severe residual regurgitation, and two developed new hemolytic anemia requiring subsequent valve surgery despite an initially successful percutaneous procedure [47]. Partial closure may therefore be inadequate when a narrow residual channel continues to generate high shear stress. By contrast, a recent technical series reported immediate elimination of the target PVL, no postprocedural hemolysis, and no residual leak at discharge except for one mild untreated secondary leak [48]. When hemolysis is the primary indication, the procedural target is near-complete elimination of the responsible jet.

Access-site and procedure-related complications remain relevant because these procedures are often long, technically demanding, and performed in elderly anticoagulated patients with multiple comorbidities. PVL closure may require prolonged catheter manipulation, complex wire externalization, repeated device exchanges, or large delivery systems in difficult anatomy. In one single-center series, the median procedure time was 110 min, and complications occurred in 9.5% of procedures, including a contained retroperitoneal hematoma, acute renal failure, and one periprocedural death after repeated unsuccessful attempts that worsened regurgitation, likely in the setting of catheter manipulation within friable tissue [49]. A transbrachial series also reported access-related and procedural complications, including a brachial pseudoaneurysm, aggravated hemolysis, and one sudden in-hospital death [50]. These observations underscore the importance of an access strategy. It influences bleeding risk, deliverability, patient mobilization, and the feasibility of the procedure in fully anticoagulated patients. The safest route is usually the one that provides the most stable trajectory to the defect with the least traumatic catheter manipulation.

Neurologic, thromboembolic, and infective complications are less frequent than device- or access-related events, but their clinical impact is greater. Patients referred for transcatheter PVL closure are often older, frail, and burdened by prior cardiac surgery, prosthetic material, chronic anticoagulation, renal dysfunction, or heart failure. In the multicenter KISS registry, in-hospital mortality after transcatheter treatment was 4.7%, while long-term stroke and endocarditis were reported in 1.8% and 1.2% of patients, respectively [42]. In another single-center registry, no cerebrovascular events were observed, but 15% of patients ultimately required valve surgery, including one urgent operation for device embolization and two operations for new hemolytic anemia [47]. Another series reported 30-day mortality of 4.7% after repeated failed closure attempts with worsening regurgitation [49]. In this setting, major adverse outcomes are seen most often after technical failure, unfavorable anatomy, or an incomplete procedural result rather than as isolated device mishaps.

Risk reduction starts before the procedure. Preprocedural imaging with transthoracic echocardiography, transesophageal echocardiography, and, when needed, cardiac computed tomography should define the location, shape, length, circumferential extent, and trajectory of the leak, together with prosthetic stability and its relation to adjacent structures [51,52]. These data guide access planning, device selection, and anticipation of possible interference with leaflet or disc motion. During the procedure, real-time 2D and 3D transesophageal echocardiography guides transseptal puncture, defect crossing, wire confirmation, device deployment, and reassessment before release [53]. Before final detachment, prosthetic motion should be checked carefully, the residual jet judged against the clinical indication, and device stability confirmed with gentle testing. When there is doubt, recapturing and repositioning are preferable to accepting a borderline result. These procedures belong in experienced valve centers with a multidisciplinary heart team and a low threshold to stop when the anatomy or procedural course becomes unfavorable.

Transcatheter PVL closure with Amplatzer-based devices offers an acceptable safety profile in experienced hands and remains an important treatment option for patients who are poor candidates for repeat surgery. The main hazards are device malposition, prosthetic interference, residual leak with persistent or new hemolysis, vascular injury, device embolization, and, less often, stroke, endocarditis, or death [42,43,44,45,46,47,48,49,50,51,52,53]. Most arise from difficult anatomy and imperfect interaction between the device and the leak channel. Safer procedures come from careful morphological planning, precise imaging guidance, appropriate sizing, and disciplined deployment. Table 3 provides a practical safety overview of the main complication domains in transcatheter PVL closure, emphasizing their clinical impact, main drivers, and preventive strategies.

## 8. Comparison with Alternative Occlusion Systems

The comparison between Amplatzer-based closure and alternative occlusion systems is best structured around three practical considerations: device geometry, deliverability, and clinical evidence. Dedicated PVL occluders were developed to improve adaptation to short or morphology-specific defects, whereas Amplatzer-based strategies are often used in a more modular way to fill or segment irregular channels. The Occlutech PLD, for example, introduced a double-disc design with square or rectangular configurations and narrow- or wide-waist variants intended to adapt more closely to short channels and discrete crescentic defects than conventional round vascular plugs [54,55]. By contrast, Amplatzer-based approaches commonly rely on one or more plugs to fill, scaffold, or segment an irregular tract, accepting that complete conformation to the entire leak geometry may not always be achievable. In practical terms, the comparison is therefore less a matter of “dedicated” versus “off-label” closure than of morphology-specific single-device sealing versus modular plug-based closure. This distinction is especially relevant because, in contemporary European practice, the two devices specifically approved for transcatheter PVL closure are the Occlutech PLD and the Amplatzer ParaValvular Plug 3, and these platforms account for most procedures across pooled observational registry experience [3].

The second major determinant is deliverability. Even when a device appears anatomically suitable, procedural success depends on whether it can be advanced, aligned, and released safely through the available access route. This remains particularly important in mitral PVL, where transseptal, retrograde, or transapical access may be required depending on leak location, channel orientation, and the degree of support needed for controlled deployment [56,57,58]. In current practice, transseptal antegrade access is used most commonly for mitral PVL, retrograde access for aortic PVL, and transapical access is generally reserved for selected cases requiring a shorter and more coaxial trajectory [3]. Device performance therefore cannot be assessed independently of access feasibility and delivery stability. Preprocedural evaluation must therefore integrate both structural and functional information before a closure system is selected.

The third consideration is the evidence base. The published experience with dedicated PVL occluders is encouraging, but it remains less extensive than the cumulative literature supporting Amplatzer-based practice. In the prospective Polish registry of the Occlutech PLD, procedural results were favorable after careful anatomy-based selection, but the study also showed that this platform was best suited to short channels and single-device closure rather than to long or highly irregular leaks. In that experience, patients were selected using meticulous real-time 3D TEE assessment, the PLD was recommended for channels shorter than 5 mm without oversizing, and the authors suggested that a multi-plug AVP III strategy may be more appropriate for long or irregular channels, whereas single-device PLD implantation may be better suited to larger, more regular defects with a short channel [55]. This distinction is clinically relevant because elongated tracts, multiple jets, and crescentic or angulated defects are common in routine practice. In such anatomies, the flexibility of Amplatzer-based approaches, including staged or multiple-plug implantation, remains an important advantage [54,55,58]. Supporting this concept, a more recent single-center registry of multiple AVP III implantation for complex PVL closure reported high technical and procedural success, frequent use of 2–4 plugs per leak, low rates of incomplete closure, and no new or worsening hemolysis during follow-up [37].

Geographic availability has also influenced adoption patterns. Dedicated PVL occluders were introduced and used predominantly in European practice, whereas Amplatzer platforms became more broadly established across centers because operators were already familiar with their delivery characteristics and could apply them across a wider range of structural interventions [55,58]. This practical familiarity has likely contributed to their continued prominence. At the same time, outcomes with dedicated systems are clinically meaningful in appropriately selected anatomies. In an international multicenter registry of the Occlutech PLD involving 136 patients across 21 sites in nine countries, functional status improved substantially, transfusion dependence related to hemolysis decreased, and the reported rates of leaflet interference, embolization, repeat closure, and valve surgery were low [24]. These findings support an important role for dedicated devices, but they should still be interpreted within the limitations of observational registry data and anatomy-based case selection.

The available literature suggests that Amplatzer-based and dedicated PVL occluders should be regarded as complementary rather than competing platforms. Dedicated devices appear particularly attractive when the leak is short, anatomically discrete, and suitable for single-device sealing, whereas Amplatzer-based strategies remain especially useful for long, irregular, crescentic, angulated, or multiple channels that may require modular closure with more than one plug [37,55]. At present, comparisons between occlusion systems are limited to observational studies and registry experience, and no randomized head-to-head study has demonstrated overall superiority of one platform across the full spectrum of PVL anatomies. Device selection therefore remains driven chiefly by morphology, access feasibility, imaging findings, and operator experience, rather than by any universal hierarchy of occlusion systems.

## 9. Evidence Gaps and Research Needs

Despite technical progress and broader procedural experience, the evidence base for transcatheter PVL closure remains limited in ways that affect daily practice. Most published data come from retrospective single-center series, multicenter registries, and nonrandomized comparative analyses, with substantial variation in patient risk profile, prosthesis type, valve position, leak mechanism, and treatment selection. Randomized trials are lacking, and current comparisons between transcatheter closure and surgery remain vulnerable to referral bias, anatomy-based selection, and differences in operator and center experience [21,42]. As a result, many decisions are still driven by local expertise and anatomical judgment rather than by a uniform comparative standard.

The first area that requires clarification is endpoint definition. Technical success, procedural success, clinical success, residual leak severity, and hemolysis response are still reported inconsistently across studies [21]. Future studies should define technical success as the successful crossing and deployment of the intended device or devices without embolization, prosthetic interference, emergency surgery, or intraprocedural death. Procedural success should add a prespecified threshold of residual regurgitation reduction and freedom from major in-hospital complications. Clinical success should be separated from procedural success and should include improvement in heart failure status, relief of hemolysis-related burden, avoidance of repeat intervention, and survival during follow-up. Without this separation, technically satisfactory procedures and clinically meaningful outcomes continue to be merged into a single, imprecise endpoint.

A second gap concerns hemolysis-specific assessment, which remains particularly inconsistent. This is a major issue because patients referred for hemolysis represent a distinct clinical subgroup, and their procedural endpoint is often different from that of patients treated primarily for heart failure. Future reports should record baseline and follow-up hemoglobin, lactate dehydrogenase, indirect bilirubin, haptoglobin, reticulocyte count, and transfusion requirement, together with the presence or absence of hemoglobinuria. Hemolysis response should be graded explicitly as complete, partial, or absent. It would also be helpful to distinguish residual leaks that are hemodynamically small from those that continue to generate high-velocity shear stress and ongoing red cell destruction [21]. That distinction is clinically relevant and is often lost when residual regurgitation is described only in echocardiographic terms.

A third priority is standardization of imaging and anatomical reporting. Current practice depends heavily on detailed echocardiographic and tomographic assessment of leak morphology, prosthetic stability, and procedural feasibility, yet imaging protocols remain variable, and interpretation remains operator-dependent. Qualification for closure usually relies on transthoracic echocardiography, transesophageal echocardiography, and, when needed, cardiac computed tomography, with attention to leak location, shape, channel length, circumferential extent, relation to prosthetic components, and hemodynamic significance [51]. Advanced image-fusion techniques may improve spatial orientation during complex procedures, but their use remains limited by technical complexity, an operator learning curve, and relatively few supporting literature [59]. Future studies should therefore report a reproducible anatomical dataset, including valve position, leak segment, channel length, maximal and minimal dimensions, circumferential involvement, angulation, number of jets, relation to leaflet or disc motion, and planned access route. This would allow for a more meaningful comparison between device platforms and procedural strategies.

A fourth gap is durability assessment. Follow-up duration varies widely, durability endpoints are not reported uniformly, and comparative long-term data against surgery remain limited. Existing reports support sustained clinical benefit in many patients after successful closure, but the literature does not yet provide a sufficiently consistent basis for comparing devices or treatment strategies over time [40,42]. Future studies should report durability using a defined set of outcomes: residual leak progression, repeat percutaneous intervention, surgical reintervention, recurrent hemolysis, heart failure hospitalization, valve-related complications, and survival. Quality-of-life measures should also be included, because symptom burden and transfusion dependence are highly relevant to this population and are not captured adequately by procedural endpoints alone.

The next phase of research should be practical rather than expansive. The field needs prospective multicenter registries built around standardized anatomical reporting, predefined procedural and clinical endpoints, and follow-up long enough to capture late failure and repeat intervention. Comparative studies should stratify patients by valve position, leak morphology, presenting syndrome, and device strategy, because a short focal aortic leak and a long crescentic mitral leak do not pose the same procedural problem. Progress will come from more disciplined reporting, anatomy-based analysis, and clearer separation of technical success, hemolysis response, and long-term durability.

## 10. Future Directions

Further progress in transcatheter PVL closure will depend on solving a limited number of persistent procedural problems rather than adding technology without a clear procedural gain. The first is device–channel mismatch. Many PVLs remain difficult to treat because the defect is short in one plane, elongated in another, angulated, or distributed along a crescentic segment of the sewing ring. Current closure often relies on operator judgment, iterative device exchange, or deployment of multiple plugs to achieve an acceptable seal. Future device development should focus on configurations that conform more predictably to irregular channels while preserving deliverability and reducing the risk of prosthetic interference [60].

A second unresolved problem is the prediction of prosthetic leaflet or disc interference before release. This remains one of the most consequential technical failures in PVL closure, particularly in mechanical valves and in defects adjacent to the hinge mechanism. Standard imaging usually defines the leak adequately, but it is less reliable in predicting how a specific device will deform within the channel and how that deformation will affect prosthetic motion. Patient-specific modeling and simulation may be useful in this setting, especially when anatomy is highly angulated or when the available landing zone is limited [61,62].

A third area is residual hemolysis after apparently successful closure. This remains a difficult problem because a hemodynamically smaller leak may still generate high shear stress if the residual channel is narrow and high velocity. For patients referred primarily because of hemolytic anemia, the procedural endpoint should not be defined only by a reduction in regurgitation grade. Future studies should examine residual jet morphology, post-procedural hemolysis markers, transfusion requirements, and the relation between device configuration and shear-producing residual flow. This would help identify anatomies in which partial closure is acceptable and those in which near-complete sealing is required.

A fourth issue is the planning of a multi-plug closure in complex anatomy. In many mitral PVLs, especially long or crescentic defects, the procedure is not simply a matter of choosing a single device, but of deciding how many devices are needed, in what sequence they should be delivered, and how they will interact once expanded. That problem is still managed largely by experience. More structured planning based on 3D transesophageal echocardiography and computed tomography could improve case selection, reduce repeated device exchange, and lower the risk of residual leak or device instability [60,61]. Automated imaging analysis may also become useful here, mainly through better segmentation, more consistent geometric measurements, and clearer communication between imaging and intervention teams rather than through autonomous decision-making [61,63].

The fifth area is the simulation of the access route and delivery feasibility. Device success depends on more than the defect itself. Access route, sheath support, crossing angle, and the ability to maintain a stable trajectory often determine whether a theoretically suitable device can be implanted safely. This is particularly relevant in mitral PVL, where transseptal, retrograde, and transapical strategies may each be appropriate depending on defect location and channel orientation. Future planning tools should therefore incorporate not only defect geometry, but also delivery path, catheter stability, and the risk of traumatic manipulation during crossing and deployment [62,63].

The near-term research agenda is practical. The field needs prospective multicenter studies with standardized definitions of technical success, clinical success, residual regurgitation, hemolysis response, prosthetic interference, and reintervention. Imaging protocols should be reported more uniformly, including the use of 3D TEE and CT for preprocedural characterization and procedural guidance. Comparative evaluation of dedicated PVL devices and modular Amplatzer-based strategies should be anatomy-specific, because short discrete defects and long crescentic channels present different technical problems. Progress in this field will come from better anatomical classification, better planning of delivery and device interaction, and clearer procedural endpoints, especially in patients treated for hemolysis rather than heart failure.

## 11. Limitations

This review has several limitations. First, it is a narrative review and does not include a formal systematic review process, meta-analysis, or structured risk-of-bias assessment. Second, the available literature is dominated by retrospective series, multicenter registries, and nonrandomized comparisons, with substantial heterogeneity in patient selection, valve position, device choice, imaging strategy, and endpoint definitions. Selection bias affects the published evidence, since patients referred for transcatheter closure are typically selected according to anatomical suitability, surgical risk, local expertise, and device availability, which limits direct comparison with surgical cohorts and may favor better procedural outcomes in experienced centers. Study-selection bias is also possible at the review level, because this review focuses on literature relevant to Amplatzer-based practice and includes both Amplatzer-specific and mixed-device cohorts, which may influence the balance of included evidence. Finally, follow-up duration and definitions of durability, hemolysis response, and clinical success vary considerably across studies, which restricts direct comparison between reports and limits the strength of pooled interpretation.

Importantly, complication rates reported in the literature may underestimate real-world risk, as most data derive from high-volume centers with advanced imaging and procedural expertise.

## 12. Conclusions

Transcatheter closure of paravalvular leak is now part of routine structural heart practice for patients with clinically significant regurgitation who face high or prohibitive risk with repeat surgery. Its role is no longer merely adjunctive in selected high-risk cases, but increasingly integrated into contemporary structural heart practice. Amplatzer-based devices remain central to this field because they provide a practical and adaptable solution for the irregular, crescentic, and elongated anatomies that characterize many PVLs. Their principal strength lies in procedural versatility, particularly in anatomies that require individualized or multi-plug strategies rather than single-device sealing.

The available evidence supports high procedural success, consistent symptom relief, and meaningful reduction in hemolysis-related burden in appropriately selected patients, although the current literature remains limited by observational study design, heterogeneous endpoint definitions, and the absence of robust comparative trials. Accordingly, current evidence supports the clinical utility of Amplatzer-based closure, but not definitive superiority over other occlusion platforms across all anatomical scenarios.

The central message of this review is that successful PVL closure depends less on the use of any single device family and more on the alignment between anatomy, imaging, access strategy, and operator expertise. In this context, Amplatzer devices are particularly valuable in anatomically complex leaks, where flexibility in device selection, repositioning, and staged or multiple-device implantation is often required. At the same time, their limitations should be recognized, especially in short, discrete, or morphology-specific defects that may be better suited to dedicated occluder systems.

Looking forward, progress in this field will depend on more standardized anatomical reporting, clearer procedural and clinical endpoint definitions, and comparative studies that stratify outcomes according to leak morphology and treatment strategy. Advances in multimodality imaging, preprocedural planning, and device design are likely to improve patient selection and procedural precision. In this evolving landscape, Amplatzer-based closure is likely to remain an important part of the therapeutic armamentarium, particularly in experienced centers able to tailor strategy to anatomy rather than force anatomy to fit a device.

## Figures and Tables

**Table 1:** Practical morphology-based selection of Amplatzer devices for PVL closure.

Device	Typical Role in PVL Closure	Best-Suited Morphology	Main Strength	Main Limitation	Typical Use Pattern
Amplatzer Vascular Plug II (AVP II)	Versatile off-label plug for routine PVL closure	Short tubular, round, or focal channels; selected elongated defects in multi-plug strategies	Low profile, good deliverability, flexible mesh	Less comfortable in broad crescentic defects; may require multiple devices	Single-plug closure in focal leaks or adjunctive use in modular closure
Amplatzer Vascular Plug III (AVP III)	Preferred Amplatzer option for complex PVL anatomy	Crescentic, oval, or elongated channels	Better adaptation to non-circular morphology	Very large or highly complex leaks may still require multiple plugs	First-line Amplatzer strategy in complex mitral or aortic PVL
Amplatzer Duct Occluder (ADO)	Selective off-label option for focal defects	Small, discrete focal channels	Familiar device, useful in anatomically suitable short channels	Poor fit in crescentic or irregular leaks; risk of residual regurgitation or prosthetic interaction	Selective use in focal leaks rather than routine first-line closure
Amplatzer Duct Occluder II (ADO II)	Adjunctive low-profile device	Small residual focal channels, especially after primary plug implantation	Very low delivery profile; useful for completion of residual jets	Limited evidence in PVL; unsuitable for broad dehiscence or elongated channels	Completion device rather than primary device
Amplatzer Muscular VSD Occluder	Bailout or selective alternative	Focal circular or moderately large defects with reliable anchoring zones	Strong anchoring and occlusive capacity	Bulkier profile; higher risk of leaflet/disc interference; poor fit for elongated leaks	Selected high-risk cases with favorable anatomy

**Table 2:** Key outcome signals from representative studies of predominantly Amplatzer-based PVL closure.

Study	Cohort/Setting	Predominant Device Strategy	Procedural Result	Clinical/Hemolysis Signal	Follow-Up/Durability Message
Calvert et al., 2016 [31]	Multicenter UK registry; mixed valve locations	Early mixed Amplatzer use, later AVP III predominant	Device implantation success 91%	Marked NYHA improvement; low rate of new transfusion-requiring hemolysis	Short- to mid-term follow-up showed sustained symptom benefit
García et al., 2017 [30]	Multicenter HOLE registry; predominantly mitral surgical PVL	Predominantly AVP III; multiple devices in 15%	Technical success 86.6%; procedural success 73.2%	No formal pooled clinical success reported	Important benchmark registry showing real-world feasibility and anatomy dependence
Smolka et al., 2017 [4]	Single-center prospective hemolysis-focused registry	AVP II/III predominant	Success assessed by >90% PVL CSA reduction rather than a single implantation endpoint	Significant LDH reduction and hemoglobin recovery; best results with near-complete residual leak reduction	Supports the importance of residual jet minimization, especially for hemolysis
Hascoët et al., 2022 [32]	International prospective multicenter registry	Predominantly Amplatzer-based mixed-device practice	Technical success 87.4% overall; higher in aortic than mitral PVL	Clinical success 70.3% in mitral and 88.0% in aortic PVL; partial transfusion benefit in hemolysis subgroup	Confirms good early results in contemporary multicenter practice
Perl et al., 2021 [33]	Single-center all-comers registry	AVP III most commonly used	Device/procedural success 88%	NYHA improvement; hemoglobin rose, LDH and transfusion need fell	Median follow-up 66 months supports medium- to longer-term benefit
Lytra et al., 2023 [34]	Multicenter Greek cohort	Amplatzer devices predominant, mainly AVP III	Technical success 90%; clinical success 78.3%	Better outcomes in heart failure than hemolysis indication; significant laboratory improvement at 1 month	Supports both symptomatic and hematologic benefits in routine practice
Sagar et al., 2022 [35]	Small single-center series of crescentic leaks	Rectangular AVP III-based strategy	Procedural success 100%	Marked NYHA improvement; hemolysis improvement not fully quantified	Suggests strong performance when device geometry is well matched to anatomy
Niikura et al., 2025 [36]	Small mitral-only case series	AVP II alone or AVP II + ADO II; mean 2.9 devices/patient	Procedural success 100%	Symptom improvement and significant LDH reduction; persistent hemolysis in a small minority	Supports modular multi-device closure in selected mitral PVL
Khanolkar et al., 2025 [11]	Prospective single-center early tertiary-center experience	AVP II predominant	Technical success 93.3%; procedural success 91.1%	Symptomatic and hemodynamic improvement reported; hemolysis benefit qualitative	Confirms feasibility of Amplatzer-dominant practice in experienced centers
Adamczyk-Filipek et al., 2026 [37]	Single-center AVP III multi-plug registry for complex PVL	AVP III exclusively; 2–4 plugs per PVL	Technical success 92.8%; procedural success 89%	No new or worsening hemolysis; low rate of clinical efficacy failure	Supports medium-term efficacy of modular multi-plug AVP III strategy

Abbreviations: ADO, Amplatzer Duct Occluder; ADO II, Amplatzer Duct Occluder II; AVP, Amplatzer Vascular Plug; CSA, cross-sectional area; LDH, lactate dehydrogenase; NYHA, New York Heart Association; PVL, paravalvular leak.

**Table 3:** Practical safety overview of transcatheter PVL closure: main complication domains, clinical impact, and prevention strategies.

Complication Domain	Typical Clinical Impact	Main Procedural/Anatomical Drivers	Key Preventive Strategy	Illustrative Signal from Representative Studies
Major adverse events	Rare but clinically serious outcomes, including procedural or early mortality	Frailty, major comorbidity burden, complex anatomy, repeated failed attempts, worsening regurgitation during the procedure	Careful patient selection, anatomy-based planning, avoidance of repeated traumatic manipulation, treatment in experienced structural centers	In-hospital mortality 4.7% in the KISS registry; 30-day mortality 4.0% with percutaneous treatment in one comparative series; 4.7% periprocedural mortality after repeated failed attempts in a single-center report [42,44,49]
Residual leak/incomplete sealing	Persistent regurgitation, limited clinical benefit, ongoing hemolysis	Elongated, multiple, or tortuous channels; incomplete device–defect match; difficult anatomy	Detailed preprocedural imaging, morphology-based device choice, cautious sizing, reassessment of residual flow before release	Moderate-to-severe residual PVL in 17.0% in the KISS registry; absent or mild residual leak in 80% in one single-center series; complete target-leak elimination in a small technical series [42,47,48]
Persistent or worsening hemolysis	Continued anemia, transfusion need, or eventual surgery despite closure	High-velocity residual jets, incomplete closure, prosthetic interference, mechanical prostheses	Aim for near-complete elimination of the responsible jet, especially when hemolysis is the primary indication	Recurrent hemolysis requiring reoperation reported in multicenter and single-center series; no postprocedural hemolysis in a small series with complete sealing [43,47,48]
Device malposition/instability	Repositioning, procedural failure, incomplete closure	Irregular anatomy, difficult tract angulation, inadequate sizing, challenging mitral anatomy	3D TEE-based planning, CT in selected cases, cautious sizing, stepwise deployment, readiness to recapture before release	In complex cases, device mismatch led to withdrawal or repositioning; simulation studies showed that initial device choice required modification in selected patients [46]
Prosthetic leaflet/disc interference	Impaired prosthetic motion, clinical failure, urgent surgery or valve re-replacement	Mechanical prostheses, defects close to leaflet/disc motion, bulky devices, inaccurate trajectory assessment	Continuous intraprocedural TEE, explicit pre-release verification of preserved prosthetic motion	3D modeling predicted leaflet blockade in selected cases; clinically significant prosthetic impingement requiring surgery reported in multicenter experience [43,46]
Device embolization	Procedural failure, hemodynamic deterioration, urgent surgery	Inadequate anchoring, poor tract stability, inappropriate device size/shape, limited delivery support	Better device–defect matching, stable wire support, careful release strategy, simulation in selected complex cases	Device embolization requiring surgery and device displacement both reported; embolization risk also highlighted in simulation-based studies [46,47,50]
Access-site/catheter-related complications	Bleeding, vascular injury, renal injury, tissue trauma, prolonged recovery	Large delivery systems, anticoagulation, prolonged procedures, repeated crossing attempts, frailty	Careful access planning, sheath minimization when feasible, streamlined strategy, real-time imaging guidance	Retroperitoneal hematoma, brachial pseudoaneurysm, acute renal failure, and catheter-related deterioration reported in single-center series [44,49,50]
Neurologic/infective complications	Infrequent but high-impact stroke or endocarditis	Comorbidity burden, prosthetic material, catheter manipulation, redo-valve population	Exclude active endocarditis, maintain strict sterile technique, use careful image-guided catheter manipulation	In the KISS registry, in-hospital stroke 1.2%, long-term stroke 1.8%, and long-term endocarditis 1.2% [42]

Abbreviations: CT, computed tomography; PVL, paravalvular leak; TEE, transesophageal echocardiography.

## Data Availability

No new data were created or analyzed in this study. Data sharing is not applicable to this article.

## Abbreviations

The following abbreviations are used in this manuscript:

ADO	Amplatzer Duct Occluder
AVP	Amplatzer Vascular Plug
CT	Computed tomography
LDH	Lactate dehydrogenase
PET	Positron emission tomography
PLD	Paravalvular leak device
PVL	Paravalvular leak
TAVR	Transcatheter aortic valve replacement
TEE	Transesophageal echocardiography
TPVLC	Transcatheter paravalvular leak closure
VSD	Ventricular septal defect
